# A combined low-frequency electromagnetic and fluidic stimulation for a controlled drug release from superparamagnetic calcium phosphate nanoparticles: potential application for cardiovascular diseases

**DOI:** 10.1098/rsif.2018.0236

**Published:** 2018-07-11

**Authors:** Alessandra Marrella, Michele Iafisco, Alessio Adamiano, Stefano Rossi, Maurizio Aiello, Maria Barandalla-Sobrados, Pierluigi Carullo, Michele Miragoli, Anna Tampieri, Silvia Scaglione, Daniele Catalucci

**Affiliations:** 1 National Research Council (CNR), Institute of Electronic, Computer and Telecommunications (IEIIT), via de Marini 6, 16149 Genoa, Italy; 2 National Research Council (CNR), Institute of Science and Technology for Ceramics (ISTEC), Faenza, Italy; 3 CERT, Center of Excellence for Toxicological Research, Department of Medicine and Surgery, University of Parma, Parma, Italy; 4 National Research Council (CNR), Institute of Genetic and Biomedical Research UOS Milan (IRGB), Milan, Italy; 5 Humanitas Clinical and Research Center, Rozzano, Milan, Italy

**Keywords:** bioreactor, cardiac cells, drug delivery, drug release, ELM stimulation, QRS complex

## Abstract

Alternative drug delivery approaches to treat cardiovascular diseases are currently under intense investigation. In this domain, the possibility to target the heart and tailor the amount of drug dose by using a combination of magnetic nanoparticles (NPs) and electromagnetic devices is a fascinating approach. Here, an electromagnetic device based on Helmholtz coils was generated for the application of low-frequency magnetic stimulations to manage drug release from biocompatible superparamagnetic Fe-hydroxyapatite NPs (FeHAs). Integrated with a fluidic circuit mimicking the flow of the cardiovascular environment, the device was efficient to trigger the release of a model drug (ibuprofen) from FeHAs as a function of the applied frequencies. Furthermore, the biological effects on the cardiac system of the identified electromagnetic exposure were assessed *in vitro* and *in vivo* by acute stimulation of isolated adult cardiomyocytes and in an animal model. The cardio-compatibility of FeHAs was also assessed *in vitro* and in an animal model. No alterations of cardiac electrophysiological properties were observed in both cases, providing the evidence that the combination of low-frequency magnetic stimulations and FeHAs might represent a promising strategy for controlled drug delivery to the failing heart.

## 1. Introduction

Cardiovascular diseases (CVDs) still represent the leading cause for morbidity and mortality in the western world [1], giving rise to many disabilities in patients. Current clinical regimens largely rely on the use of drugs, medical devices and changes in lifestyle but, despite large beneficial outcomes, none of these treatments is as yet fully efficient in preventing the progression of the pathology. In this context, the identification of alternative therapeutic approaches is urgently needed.

Encouraging results come from the emerging field of nanomedicine, which, through the development of innovative therapies based on nanoparticles (NPs), aims to ameliorate the current approaches for the delivery of therapeutic agents [2,3]. In this context, the use of NPs can help to augment the payload protection against an immediate degradation in the bloodstream, to increase local drug release thereby minimizing systemic side effects, and to improve treatment efficacy and cell selectivity [4,5]. In this domain, we have recently demonstrated that the administration of NPs through inhalation is an effective method to target the heart and a promising pharmacological treatment of heart failure [6]. Additionally, to improve therapeutic efficacy, one of the most fascinating approaches consists of the use of stimuli-responsive NPs providing an on-demand release of bioactive molecules under the action of an external stimulus [7]. Among these, the use of an alternating electromagnetic field (EMF) is becoming highly attractive over others, as it allows deeper penetration in the body and a less harmful ionizing effect compared to near-infrared light and X-ray radiations, respectively [8].

Magnetic field-responsive NPs (MNPs), particularly those based on iron oxides, hold great promise for their application in the therapeutic and diagnostic fields [9], and owing to their unique magnetic properties, appropriate methods and devices can be designed for their *in vitro* and *in vivo* manipulation [4,10–12]. Owing to their inherent low toxicity and easy synthesis, superparamagnetic iron oxide NPs (SPIONs), consisting of mixtures of magnetite, maghaemite and haematite, are the most studied and used MNPs for biomedical applications [13]. However, despite their clinical use being approved by the American Food and Drug Administration (FDA), SPIONs have been recently associated with several concerns about their long-term cytotoxicity. Recent studies reported that their chronic administration could lead to the accumulation of a high quantity of iron in the liver and kidney, causing an imbalance in their homeostasis as well as cytotoxic and inflammatory effects [14]. Owing to these restrictions, new forms of biocompatible and biodegradable MNPs with a good magnetic moment, drug-loading capability and low iron content have been studied. Recently, superparamagnetic Fe^2+^/Fe^3+^-doped calcium phosphates (CaPs) in the crystalline form of hydroxyapatite (HA) (hereafter called FeHA) have been developed [15,16] Owing to their chemical resemblance with the inorganic phase of human mineralized tissues (i.e. bone and dental tissue), CaPs-based materials are the most widely accepted biocompatible and biodegradable compounds for different biomedical applications spanning from nanomedicine to bone tissue engineering [17–19]. Interestingly, it was reported that the iron atoms of FeHAs display a higher magnetic moment with respect to those of maghaemite and magnetite NPs [15]. It was also demonstrated that the application of a low-pulsed magnetic field triggers the release of therapeutic molecules (i.e. doxorubicin) from FeHAs by fostering their mechanical shacking and the consequent detachment of drug from their surface [7].

Despite the progress in the synthesis of biocompatible MNPs [20], the effects of the application of external EMF on biological systems still remain a major concern, highlighting the strong need for the generation of proper stimulating magnetic/electro-magnetic bioreactor devices (MEBDs) for *in vitro* cell biology studies as well as for *in vivo* tests in animal models [21,22]. MEBDs typically consist of commercial generators of pulsed EMF, which induce an EMF between two solenoids (i.e. Helmholtz coils) [23–27]. Owing to the variety of experimental set-ups and MNPs, the application of such devices is still associated with contradictory results, leading to the critical need for further *in vitro* and *in vivo* studies [28–30]. Furthermore, with a future view of setting up a treatment for the cardiovascular system, the magnetically induced process of drug release could be affected by the fluidic stimuli, which are not negligible. Thus, the synergistic effect of fluidic and magnetic forces acting on MNPs constitutes an additional feature to be considered.

The aim of this work was to evaluate whether the release of drugs from FeHAs can be magnetically triggered by a custom-made low-pulsed MEBD, which is fully integrated with a fluidic system. Moreover, the compatibility of FeHAs and the MEBD-generated electromagnetic exposure to the cardiac system have been systematically evaluated *in vitro* and *in vivo*.

## 2. Material and methods

### 2.1. Synthesis and characterization of FeHAs

Superparamagnetic iron-substituted hydroxyapatite nanoparticles (FeHAs) were synthesized as previously reported by Tampieri *et al*. [15]. Briefly, an aqueous solution of H_3_PO_4_ (44.4 g in 300 ml) was dropped into an aqueous suspension of Ca(OH)_2_ (50.0 g in 400 ml) containing FeCl_2_4 · H_2_O (12.7 g) and FeCl_3_ · 6H_2_O (17.9 g) as sources of Fe^2+^ and Fe^3+^ ions, respectively, under constant heating and stirring at 40°C. Once the neutralization reaction was completed, the solution was kept under the same conditions used during the neutralization reaction for 1 h, and then left to age for 24 h at room temperature without further stirring. The precipitate was separated from the mother liquor by centrifugation, washed three times with bi-distilled water by centrifugation and then freeze-dried.

Iron-free hydroxyapatite NPs (HAs) were also prepared as a comparison. The synthesis was carried out by simple neutralization of the Ca(OH)_2_ without adding any iron precursor and following the same conditions as used to synthesize FeHAs (e.g. temperature, Ca/P ratio, etc.).

The morphology of NPs has been evaluated by transmission electron microscopy (TEM) observations using a FEITecnai F20 instrument equipped with a Schottky emitter and operating at 120 keV. Samples were dispersed in isopropyl alcohol and a droplet of the suspension was evaporated at room temperature and under atmospheric pressure on a holey-carbon film.

The size and ζ-potential of NPs were measured by dynamic light scattering (DLS) using a Zetasizer Nano ZS (Malvern, Worcestershire, UK). NPs were suspended in 0.01 M HEPES buffer at pH 7.4 at a concentration of 0.5 mg ml^−1^. For size distribution measurements, low-volume quartz cuvettes (Hellma, Mullheim, Germany) were used. Ten runs of 30 s were performed for each measurement and four measurements were carried out for each sample.

### 2.2. *In vitro* tests on FeHAs

#### 2.2.1. Cell culture

HL-1 cardiac cells were grown in Claycomb medium (Sigma-Aldrich) supplemented with 10% FBS (Sigma-Aldrich), 1% of penicillin-streptomycin (Pen-Strep 10000 U/mi, Lonza), 1% of ultraglutamine 1 (200 mM, Lonza) and 1 mM of norepinephrine (Sigma-Aldrich) in gelatin/fibronectin precoated T75 flasks. At full confluence, cells were split 1 : 3 according to Claycomb's instructions [31].

#### 2.2.2. Cell viability analysis

The effect of FeHAs on HL-1 cell viability was analysed using a RealTime-GloMT luminescent kit (Promega) according to the manufacturer's instructions. Briefly, 5 × 10^4^ cells well^−1^ were seeded in precoated white-walled 96-well plates (1 h, 37°C). Twenty-four hours after plating, HL-1 cells were treated with various concentrations of FeHAs. To continuously monitor the viability of treated HL-1 cells in real time, RealTime-Glo Reagents were added at the same time as FeHAs. Luminescence intensity at the desired time points, indicated in the text, was measured up to 48 h using a microplate reader SynergyTM H4 Hybrid Multi-Mode Microplate Reader (BioTek). Three biological replicates were analysed in triplicate.

#### 2.2.3. Reactive oxygen species measurement

HL-1 cells were seeded at 1 × 10^4^ cells well^−1^ density in precoated white-walled 96-well plates (1 h, 37°C). Twenty-four hours after plating, different doses of FeHAs were administered as indicated in the text. Levels of reactive oxygen species (ROS) were detected after 24 h of FeHA treatment using non-lytic ROS-Glo™ H_2_O_2_ Assay (Promega) according to the manufacturer's instructions. The luminescent signal was measured 2 h after incubation with H_2_O_2_ substrate and after 20 min incubation with ROS-GloTM reagent with a microplate reader, SynergyTM H4 Hybrid Multi-Mode Microplate Reader (BioTek). Three biological replicates were analysed in triplicate.

#### 2.2.4. Viability, cytotoxicity and caspase 3/7 assay

HL-1 cells were seeded at a density of 1 × 10^4^ cells well^−1^ in precoated black-walled 96-well plates (1 h, 37°C). Twenty-four hours after plating, different doses of FeHAs were administered as indicated in the text. Viability, cytotoxicity and caspase-3/7 activities were measured after 24 h of FeHA treatment using the ApoTox-Glo triplex Assay (Promega) according to the manufacturer's instructions. Fluorescence at 485Ex/520Em and bioluminescence reactions were measured using a microplate reader, SynergyTM H4 Hybrid Multi-Mode Microplate Reader (BioTek). Three biological and technical replicates were carried out.

#### 2.2.5. Inhibition of endocytosis

Clatrin (PitStop2, Abcam) and dynamin (MiTMAB, Abcam) inhibitors were used at 1 µM each. After a 30 min incubation at 37°C in serum-free conditions, cells were treated with different doses of FeHAs as indicated in the text.

### 2.3. *In vivo* experiments on FeHAs

The study population consisted of 12 male Sprague Dawley rats bred in our departmental animal facility, aged 12–14 weeks and weighing 300–350 g. This study was carried out in accordance with the recommendations in the Guide for the Care and Use of Laboratory Animals of the National Institute of Health (Bethesda, MD, USA, revised 1996). The protocol was approved by the Veterinary Animal Care and Use Committee of the University of Parma (Permit: PMS 53/09) and conforms to the National Ethical Guidelines of the Italian Ministry of Health (Permit: PMS 53/09). All effort was made to minimize suffering.

The animals were separated and placed in special cages until the start of the experiments, in a temperature-controlled room at 20–24°C, with the light on between 7.00 and 19.00. The bedding in the cages consisted of wood shavings; food and water were freely available.

To evaluate the effect of FeHAs and HAs on the electrophysiological properties of rat heart *in vivo,* we performed epicardial potential measurements by means of an 8 × 8 electrode array [32]. Rats were anaesthetized with a mixture of 40 mg kg^−1^ip ketamine chloride (Imalgene, Merial, Milano, Italy) and 0.15 mg kg^−1^ ipmedetomidine hydrochloride (Domitor, Pfizer Italia S.r.l., Latina, Italy). After anaesthesia, a 16-gauge catheter was gently inserted into the trachea of rats in order to deliver approximately 50 µl of saline solution +2 mg kg^−1^ NPs (either FeHAs or HAs) by means of a laboratory bench P200 pipette (Gibson, UK) [33]. After waking (administration of 0.15 mg kg^−1^atipamezole hydrochloride; Antisedan, Pfizer, Italy), rats were left conscious for 4 h before performing *in vivo* electrophysiological recordings. After a second anaesthesia administration and under artificial respiration, the heart was exposed through a longitudinal sternotomy and suspended in a pericardial cradle. In the present study, an 8 × 8 row and column electrode matrix with 1-mm inter-nodal resolution was fabricated from surgical cotton gauze and was positioned in order to cover part of the anterior surface of the right (RV) and left (LV) ventricles. To evaluate spatial change in excitability, we measured the threshold intensity for a stimulus of 1 ms duration in 13 different points in each animal. An activation sequence (isochrone map) was determined from the activation times of paced beats, where the conduction velocity was computed longitudinally (CVl) and transversally (CVt) to epicardial fibre orientation.

### 2.4. Bioreactor design and development

A MEBD with adjustable features (i.e. duty cycle, waveform, maximum stimulation amplitude, etc.) based on the Helmholtz coils configuration was fabricated for the generation of an intense and spatially homogeneous EMF. Firstly, preliminary theoretical modelling was performed to optimize the magnetic density by tuning the geometrical parameters (i.e. radius of the coils and distance between the coils) and the physical cues (i.e. number of turns, diameter of copper threads) of solenoids. To this aim, simulation of the magnetic flux density within the two coils was performed by using the Matlab platform (Version 2016a).

Technical details of the electro-stimulating bioreactor are reported in the electronic supplementary material.

Subsequently, a custom-made polyvinylchloride holder was realized to host the Helmholtz coils without interfering with the field generated. Between the two coils a poly(methyl methacrylate) (PMMA) planar support was created to host Petri dishes (60 mm diameter) for cell cultures.

Specifically, each coil is made of 50 turns of 1.18 mm enamelled copper wire and with a radius (a) and distance (h) equal to 50 mm. A digital Hall effect Gaussmeter (probe UGN3503U) was used to measure the EMF created between the coils.

Measurements of the circulating current and the intensity of B, for sin signals, were performed to test the uniformity of the EMF for the entire height within the coils.

In particular, the following parameters were set: amplifier gain = 1 dB, peak-to-peak amplitude Vpp = 2, and sin function, obtaining a current value of 4.76 ± 0.06 A and a derived EMF intensity of 3.27 ± 0.68 mT in the frequency range of 15–100 Hz. These parameters have been set in the following experiments.

### 2.5. Drug loading and release experiments

FeHAs or HAs loaded with ibuprofen (IBU) (used as a model drug) were prepared by mixing NPs (20 mg) with 5 ml solutions of IBU (10 mg ml^−1^) dissolved in KCl 0.01 M. All the loading experiments were carried out at 37°C. The amount of IBU loaded on NPs was estimated as the difference between the initial concentration of IBU and that at the end of the adsorption experiments as determined by UV-Vis spectrometry (Cary Bio spectrophotometer, Varian, Palo Alto, USA). For this purpose, samples were centrifuged (5000 r.p.m. for 5 min) to retrieve the solid followed by three washings with water by centrifugation and freeze-dried, and the concentration of IBU in the supernatant was determined from the adsorption intensity at *λ* = 266 nm (ɛ = 6600 M^−1^ cm^−1^). The conditions used for the loading were optimized analysing the adsorption kinetics as well as the adsorption isotherms (see the electronic supplementary material).

Suspensions of FeHAs and HAs (0.67 mg ml^−1^) loaded with 0.2 mg IBU per milligram of NPs were injected into a fluidic circuit of citrate buffer (0.1 M, pH 6.0) under coupled magnetic and fluidic stimulations, mimicking the cardiovascular environment (velocity of the flow = 5.7 cms^−1^). The fluidic circuit was placed inside the electromagnetic stimulation apparatus and the drug release efficacy was tested at different regimes of EMF, varying the frequencies of magnetic stimulation (15, 75, and 100 Hz). The effect of the fluidic stimulation decoupled from the electromagnetic one was also assessed. In brief, after 5 and 120 min, the circulating solution was collected and absorbance readings at 266 nm were assessed spectrophotometrically. The chemical degradation of FeHAs and HAs under the same experimental conditions was evaluated as the extent of Ca, P and Fe released in the flowing solution. Quantification was carried out by an inductively coupled plasma optical emission (ICP-OES) spectrometer (Synchronous Vertical Dual View (SVDV) 5100, Agilent Technologies, USA). Supernatants were collected after 2 h under fluidic and magnetic stimulations and dissolved in 1 wt% ultrapure nitric acid. The analytical emission wavelengths were: Ca 422.673 nm, P 213.618 nm and Fe 259.940 nm. Each measure was recorded three times for statistical reliability.

### 2.6. Measurement of heat dissipated from the FeHAs under magnetic stimulation induced by a magnetic/electro-magnetic bioreactor device

A theoretical model was developed to evaluate the eventual heat dissipation due to the electromagnetic stimulation of FeHAs. In particular, theoretical simulations have been performed by using the Matlab platform (v. 2016a). Considering *μ*_0_ the magnetic permeability in the vacuum, *X*_0_ the magnetic susceptibility, *H*_0_ the EMF strength and *f* the frequency of stimulation, the dissipated heat was calculated following the density power law here reported:2.1
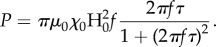


The model was then experimentally validated. NPs were subjected to different EMFs (the same used in the previous section) and the related temperature variations were measured with an infrared camera. A thermographic camera (320 × 240 pixel, Flir A315, Italy) was positioned above the coils via a photographic easel, which allowed movement along the three axes. The NPs were injected within the fluidic circuit and different EMFs were applied, while a laptop connected with the thermographic camera showed an infrared video in real time representing the temperature variations.

### 2.7. Isolation of adult mouse cardiomyocytes and culture under electromagnetic field stimulation

Adult mouse CMCs were isolated from 8-week-old male mice (C57B6/J) according to previously published protocol [34]. Mouse procedures were performed according to institutional guidelines in compliance with national (4D.L. N.116, G.U., suppl. 40, 18-2-1992) and international law and policies (EEC Council-Directive 86/609, OJ L 358,1,12-12-1987; NIH Guide for the Care and Use of Laboratory Animals, US National Research Council-1996 and new directive 2010/63/EU). The protocol was approved by the Italian Ministry of Health. Special attention was paid to animal welfare and to minimize the number of animals used and their suffering.

The apparatus for the electromagnetic stimulation was positioned on the optical microscope for biological validation. The same range of magnetic stimulation frequencies used to test the drug release efficacy was set. In particular, serial EMF stimulations at 15, 75, and 100 Hz of frequency, each one prolonged for 40 s, were performed and repeated for each CMC selected from each sample. EMFs and current values were measured and recorded both before and after each experiment. The sham group was treated like the experimental group, except for EMF exposure. Measurements were made by independent researchers. No temperature difference was observed between exposure and sham coils during the exposure. The experimental group was exposed to a current of 4.76 ± 0.06 A flowing in the circuit, generating an EMF of 3.27 ± 0.68 mT. Each experiment was carried out in triplicate, and for each animal 10–15 cells were analysed *in vitro*.

### 2.8. Cardiomyocyte calcium transients and contraction measurements

The electromagnetic stimulator system was interfaced with an epi-fluorescence inverted microscope (Nikon, Eclipse TE2000-S) with the CCD camera (IonoptixMyoCam, Ionoptix, Milton, MA) for parallel measurements of CMC sarcomere length and Ca^2+^ transients, which were carried out as elsewhere described [35]. In brief, CMCs, previously loaded with 1 µM of the Ca^2+^ probe Fura-2 AM (Life technologies) were placed in a perfusion system and continuously perfused with a standard Tyrode solution containing 1.2 mM Ca^2+^. The loaded cells were field stimulated (20 V, 4 ms) at a frequency of 0.5 Hz and subjected to electromagnetic stimulations as described above. Sarcomere length and Fura-2 ratio (measured at 512 nm upon excitation at 340 and 380 nm) were simultaneously recorded. Data were finally analysed using the Ion Wizard software (IonOptix Corp., Milton, USA) and parallel measurements of CMC contraction and calcium transients derived.

### 2.9. *In vivo* electromagnetic field exposure on rats

The study population consisted of five male Sprague Dawley rat, weighing 280–300 g. Animals were anaesthetized with a mixture of ketamine chloride 40 mg kg^−1^ IP (Imalgene, Merial, Milano, Italy) and medetomidine hydrochloride 0.15 mg kg^−1^ (Domitor, Pfizer Italia S.r.l., Latina, Italy), and all efforts were made to minimize suffering.

To note, the physiological heart rate was 300 beats per minute (bpm), whereas under anaesthesia it ranges 180–240 bpm. In the left hind leg, we performed a small incision of the skin for the subcutaneous insertion of a spiral-shaped electrode (ground electrode), while two small incisions were made in the suprasternal and xiphoid region for the subcutaneous insertion of the recording electrodes. Each animal was placed supine on the PMMA support having small holes on each side. A lanyard was placed both under the teeth to keep the head firm and around the rat's feet and tied to the PMMA surface to minimize discomfort to the animal. The PMMA where the rat was laid was positioned on top of the bottom coil and two pieces of foam rubber (3.5 cm) were placed as a support holding the top coil.

The experiment consisted in recording an (ECG) of the rat hearts under application of a low-frequency EMF to determine whether it caused any changes to the cardiac frequency (RR interval) and/or to ventricular activation duration (QRS complex). The EMF was generated by a sinusoidal wave having a Vpp = 2 and the amplifier gain was set to 1 dB. The frequency was varied up to 15 Hz (3, 5, 10, 13, and 15 Hz). The current flowing in the circuit was 3.34 ± 0.075 A, the EMF was 4.03 ± 0.086 mT. The surrounding field was measured to be 0.0096 mT. Each set-up sequence was recorded three times for statistical reliability.

The RR interval and the QRS complex were acquired using a MUX integrated system (Crescent Electronics, USA) and analysed by custom-made software [32]. QRS complex and RR interval measurements were acquired three times for each experimental pattern (single frequency, multiple continuous frequency and multiple discontinuous frequency recording). The central measurement set was chosen for statistical analysis. The heart rate was 180 bpm on average. RR interval and QRS complex durations were evaluated every 6 s to take into account a possible longitudinal variation in time during each 30 s long stimulation (normal (no stimulation), 3, 5, 10, 13, and 15 Hz).

### 2.10. Statistical analysis

For *in vitro* tests of FeHAs, statistical significance was determined by ANOVA Tukey's *post hoc* multiple comparisons test. For *in vivo* tests of FeHAs, correlation analysis was performed using the one-way ANOVA. For drug release tests, statistical analysis was assessed using Student's paired *t*-test. For cardiomyocyte calcium transients and contraction tests, statistical analysis was performed by ANOVA. For *in vivo* EMF exposure on rat tests, correlation analysis was performed using the parametric Student *t*-test. For all the analysis the differences between datasets were considered significant at *p* < 0.05. All data were analysed by Prism 6.0 (GraphPad Software, Inc.).

## 3. Results

### 3.1. Chemical–physical features of FeHAs

FeHAs have essentially been characterized in a previous work [16]. According to the earlier reported magnetic characterizations, FeHAs display typical behaviour of superparamagnetic NPs without a residual magnetization at room temperature and with mass magnetization at saturation of 8.7 emu g^−1^ after subtraction of the paramagnetic contribution [16,36]. Details of the mechanisms occurring at the nano/atomic scale in determining the unusual magnetic features of FeHAs which exhibit a very high net magnetic moment per iron atom equal to 130 emu g^−1^ of Fe have been described elsewhere [16]. FeHAs are also endowed with a hyperthermia feature, corresponding to a temperature increase of about 40°C in 60 s under an alternating EMF of 0.03 N A^−1^ m^−1^ at a frequency of 293 kHz [15]. Superparamagnetic FeHAs present needle-like crystals with dimensions of 70–100 nm in length and 15–25 nm in width that are composed of smaller aggregated particles of about 5–10 nm in width and 10–20 nm in length (electronic supplementary material, figure S1a). The TEM micrograph also revealed the presence of electron-dense round-shaped NPs (5–10 nm in size) on the surface of the needle-like NPs (electronic supplementary material, figure S1b). In the work of Iannotti *et al*., these ‘dark’ NPs were identified as maghaemite by Mossbauer spectroscopy, extended X-ray absorption fine structure (EXAFS) spectroscopy and electron diffraction [16]. The formation of maghaemite as the secondary phase is due to the combination of two mechanisms occurring during FeHA synthesis: (i) Fe^2+^ oxidation and (ii) oxidation of magnetite formed by co-precipitation. HAs display similar morphology and dimensions to FeHAs, but obviously no external Fe-rich phases are observed (electronic supplementary material, figure S1c). The mean hydrodynamic radius (R_H_) and ζ-potential of FeHAs and HAs, calculated by DLS, were 205 ± 2 nm and 120 ± 2 nm (with a polydispersity index of 0.3 and 0.2, respectively) and −22.0 ± 0.8 mV and −15.7 ± 1.2 mV, respectively (electronic supplementary material, figure S2).

### 3.2. *In vitro* and *in vivo* biocompatibility of FeHAs

**Figure 1.**
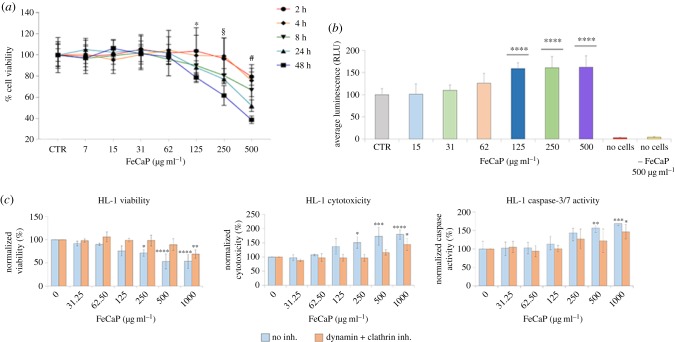
Biocompatibility of FeHA to HL-1 cardiac cells: effects on cell viability, caspase 3/cytotoxicity and ROS production. (*a*) Cell toxicity as measured by the Real Time Glo assay in HL-1 cells treated as indicated during 48 h. Data are presented as mean ± s.d.; *n* = 9, in three independent experiments for each experimental condition. Using one-way ANOVA and Tukey's *post hoc* test, * indicates *p* < 0.05 significance for the 125 µg ml^−1^ FeHA dose after 48 h, § indicates *p* < 0.05 significance for the 250 µg ml^−1^ FeHA dose after 8, 24 and 48 h, and # indicates *p* < 0.01 significance for the 500 µg ml^−1^ FeHA dose after all timing treatments: 2, 4, 8, 24 and 48 h compared with control non-treated cells (CTR). (*b*) ROS production was used to evaluate FeHA-induced oxidative stress in HL-1 cardiac cells after 24 h treatment. Data are presented as mean ± s.d.; *n* = 9, in three independent experiments for each experimental condition. ****p < 0.0001 indicate significance calculated for each FeHA dose compared with CTR using one-way ANOVA and Tukey's *post hoc* test. (*c*) Correlation of viability, cytotoxic and apoptotic levels’ detection via the activated caspase 3/7 assay in HL-1 cells pretreated as indicated with clathrin and dynamin inhibitors and then with increasing concentrations of FeHA during 24 h. Data are presented as mean ± s.d.; *n* = 9, in three independent experiments for each experimental condition. *p < 0.05, **p < 0.01, ***p < 0.001 and ****p < 0.0001 indicate significance for each FeHA dose compared with CTR using two-way ANOVA and Tukey's *post hoc* test.

To investigate the potential cytotoxic effects of FeHAs, HL-1 cardiac cells were treated with incremental doses of FeHAs (0–500 µg ml^−1^) and viability analysed using a bioluminescent assay. As shown in figure 1*a*, HL-1 cells largely tolerated an acute administration of FeHAs showing more than 90% viability up to 125 µg ml^−1^ of FeHAs at 24 h post-administration. On the other hand, as expected, a cytotoxic effect was detected only when the FeHA concentration was raised to significantly higher doses (greater than 250 µg ml^−1^), determining detrimental effects starting a few hours post administration.

ROS production is a frequently reported cytotoxicity associated with changes in structural and physico-chemical properties of NPs [37]. However, we observed that the FeHA-mediated induction of ROS in HL-1 cells is limited, reaching a significant level only at the concentration of 125 µg ml^−1^ (figure 1*b*).

To further characterize FeHA biocompatibility and assess whether early stages of apoptotic processes might be induced upon its administration [38], HL-1 cells were subjected to incremental doses of FeHA during 24 h and an analysis of viability, cytotoxicity and caspase 3/7 activity was performed at 24 h post-treatment. Confirming the above data, HL-1 cells largely tolerated acute administration of FeHAs without showing any effect in terms of viability and sign of apoptosis and cytotoxicity up to 125 µg ml^−1^ (figure 1*c*). To determine whether the cellular internalization was due to active mechanisms of endocytosis, specific inhibitors of clathrin and dynamin, which are involved in the initial processes of endocytosis and invagination from the plasma membrane [37], were used. Following short-duration pretreatment with these inhibitors, HL-1 cells were exposed to increasing concentrations of FeHAs and assays for viability, cytotoxicity and caspase 3/7 activity were performed as described above. In accordance with previous studies of negative-charged NPs [4,39], the inhibition of dynamin/clathrin-mediated endocytosis significantly reduced cellular toxicity at high doses of FeHA (250 and 500 µg ml^−1^), suggesting that internalization occurs via conventional clathrin- and dynamin-mediated endocytosis (figure 1*c*).

Collectively, our data show relevant biocompatibility of FeHAs to levels comparable to that of iron-free CaPs previously reported [4].

Following the above *in vitro* characterization, we evaluated the effects of FeHAs and HAs on the electrophysiological properties of rat heart *in vivo.* Epicardial mapping, performed on animals treated with a single intratracheal instillation of saline solution +2 mg kg^−1^ NPs (either FeHAs or HAs), pointed out a similar behaviour (table 1). In detail, cardiac excitability evaluated as the threshold intensity for a stimulus of 1 ms duration was similar between the two groups (FeHA: 51.9 ± 5.4 µA versus HA: 42.5 ± 2.9 µA; *p* = 0.11). In addition, longitudinal and transverse ventricular conduction velocities as well as the anisotropy ratio were not significantly different between the two groups. Specifically, CVl was 0.63 ± 0.02 m s^−1^ in the FeHA group while being 0.60 ± 0.01 m s^−1^ in the HA group (*p* = 0.10), and CVt was 0.28 ± 0.01 m s^−1^ in the FeHA group while being 0.29 ± 0.01 m s^−1^ in the HA group (*p* = 0.40). The anisotropy ratio was 2.27 ± 0.07 in the FeHA group and 2.09 ± 0.05 in the HA group (*p* = 0.053). Altogether, these data show good *in vivo* biocompatibility of FeHAs.

**Table 1.** Epicardial mapping parameters in both experimental groups. All values are reported as mean ± s.e.m. CVl: longitudinal conduction velocity. CVt: transversal conduction velocity.

	threshold (µA)	CVl (m s^−1^)	CVt (m s^−1^)	anisotropy ratio
FeHA	51.9 ± 5.4	0.63 ± 0.02	0.28 ± 0.01	2.27 ± 0.07
HA	42.5 ± 2.9	0.60 ± 0.01	0.29 ± 0.01	2.09 ± 0.05

### 3.3. Development of magnetic/electro-magnetic bioreactor device controlling drug release from FeHAs

Upon assessments of FeHA biocompatibility *in vitro* and *in vivo*, we proceeded with the development of a novel custom-made MEBD to control the kinetics of drug release, by tuning the frequencies of magnetic stimulation applied to MNPs.

**Figure 2.**
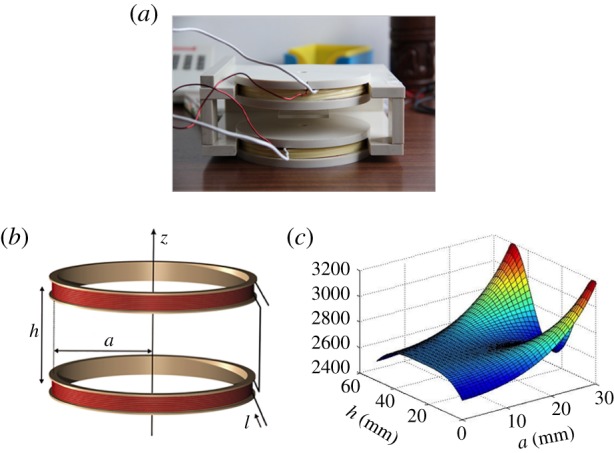
Picture (*a*) and schematic representation (*b*) of the Helmholtz coils. Magnetic induction modulus profile within coils (*c*); the warm colours (red and yellow) indicate generally higher values in terms of field strength, while cold ones (blue) lower values.

**Figure 3.**
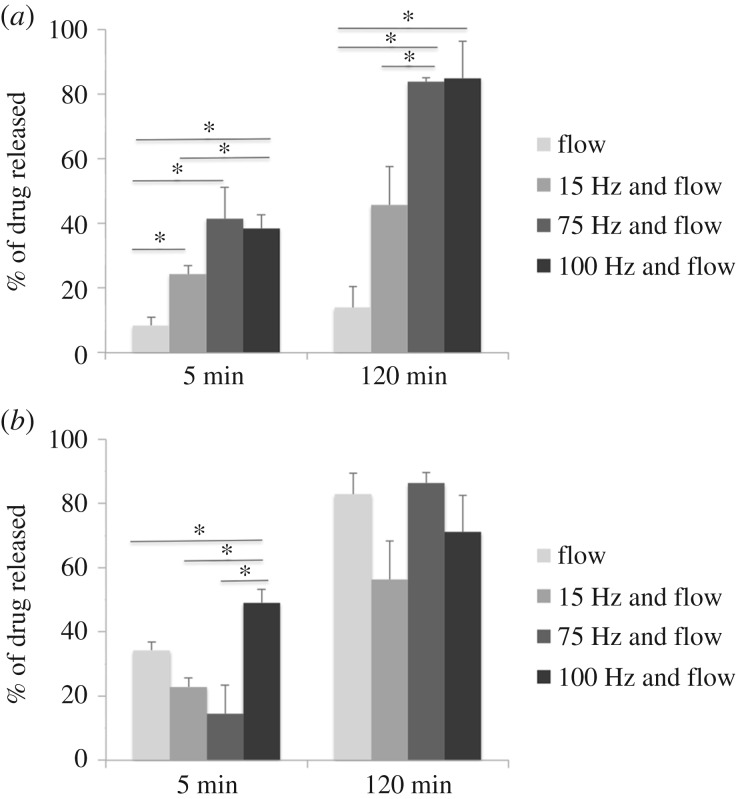
Percentage of IBU released within the fluidic circuit under magnetic and fluidic stimulation from FeHA (*a*) and HA (*b*) NPs at different time points. Values are reported as mean ± s.d., * indicates *p* < 0.05 significance.

Helmholtz coils were positioned within a polyvinylchloride holder (figure 2*a*), which allowed within the working region between coils (figure 2*b*) an appropriate spatial homogeneity and EMF intensity for *in vitro* and *in vivo* testing (figure 2*c*). To validate the system, we performed experiments on NPs loaded with IBU, a tool drug that due to its distinct spectrophotometric properties facilitates the monitoring of the release. In addition, to mimic the cardiac circulatory environment and assess whether a fluidic environment might affect the drug release, a fluidic circuit system was integrated to the device. FeHA and HA suspensions (0.67 mg ml^−1^) loaded with 0.2 mg of IBU per mg of NPs were injected separately within the fluidic circuit and subjected to magnetic and fluidic stimulation. In this setting, the drug release over time was investigated at different regimes of EMF (figure 3). The application of a pulsed EMF on FeHA suspension enhanced the efficacy of drug release, which was proportional to the frequency of the applied EMF (figure 3*a*). No evident effect of the EMF and/or fluidic environment on the IBU released from HAs was observed, both after 5 and 120 min (figure 3*b*). A higher IBU release was recorded from HAs with respect to FeHAs when exposed to fluid flow. This effect is probably due to the higher affinity of IBU for FeHA than HA, as evaluated by studying the adsorption kinetics and adsorption isotherms and their fitting according to the Sips model (electronic supplementary material, figures S3 and S4). The amounts of Ca, P and Fe released from FeHAs and HAs under fluidic and magnetic stimulation are reported in table 2. Values were quantified to assess the effects of both the impulses on NPs and can be considered as an index of their dissolution. In the first place, it is worth noticing that the quantity of Ca and P released from both NPs are comparable, but are much higher than those reported in a similar study [7] on FeHAs using an equivalent pulsed magnetic stimulus (2.0 mT at 75 Hz) in no-flux conditions. Thus, here we ascribe the observed faster dissolution of NPs to the applied flux used to mimicking the physiological arterial–venous blood flow. No significant differences were observed between the amounts of Ca, P and Fe released with the increasing amplitude of the magnetic stimulus. These results clearly indicate that the observed increase in the quantity of released IBU was triggered by higher frequencies of the magnetic stimulus rather than by a faster degradation of the carriers.

**Table 2.** Released mass percentages of calcium, phosphorous and iron from FeHA and HA NPs after 120 min under fluidic and electromagnetic stimulation determined by ICP-OES. Values are reported as the mean ± s.d.

	flow	flow+15 Hz EMF	flow+75 Hz EMF	flow + 100 Hz EMF
Ca release (wt%)				
HA	63.6 ± 3.6	64.0 ± 2.3	64.9 ± 5.5	68.4 ± 5.5
FeHA	63.4 ± 2.8	67.9 ± 5.1	68.3 ± 4.1	70.9 ± 2.1
P release (wt%)				
HA	64.2 ± 5.8	65.2 ± 3.2	66.7 ± 8.2	69.2 ± 5.3
FeHA	63.5 ± 6.2	70.9 ± 8.2	71.2 ± 7.6	73.7 ± 4.1
Fe release (wt%)				
HA	—	—	—	—
FeHA	26.8 ± 0.8	32.0 ± 4.3	32.0 ± 2.6	34.5 ± 2.9

### 3.4. Measurement of heat dissipated from the FeHAs under a magnetic/electro-magnetic bioreactor device

**Figure 4.**
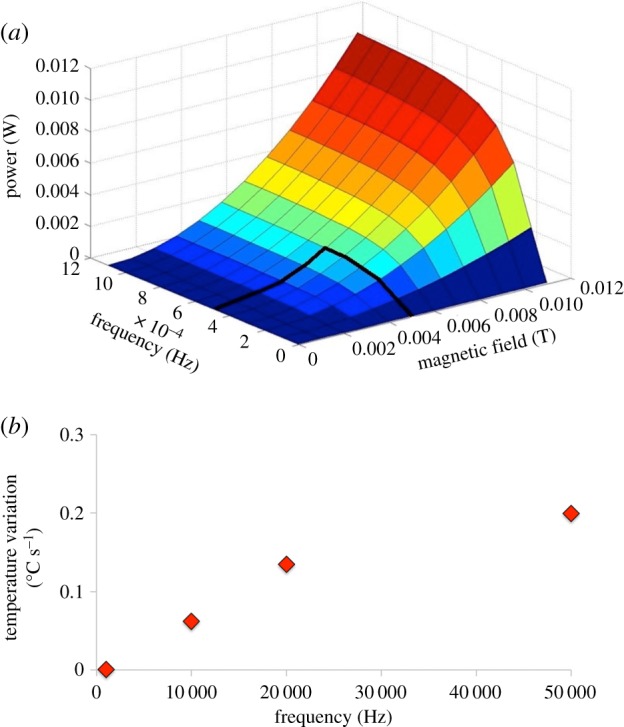
Representation of the electromagnetic stimulation parameters (frequency of stimulation and magnetic field intensity) necessary to induce heat dissipation (*a*) and correlation between temperature variations and frequency of stimulations considering *B* = 3.27 mT (*b*).

The results obtained from the theoretical modelling related to the heat dissipation during the electromagnetic stimulation of FeHAs are summarized in figure 4. Our data show that the combination of the frequency of stimulation and the EMF is necessary to induce heat dissipation (figure 4*a*). In particular, the temperature increase occurs in the red areas represented in the graph, while the blue areas are not subjected to temperature variations. Notably, the graph shows that heat dissipation does not occur by setting the EMF employed in this work; therefore heat does not induce the drug release from the NPs (frequency of stimulation up to 100 Hz; EMF: 3.27 ± 0.68 mT).

The temperature variations in terms of degrees Celsius over seconds (°C s^−1^) are derived and plotted in figure 4*b*. Considering *B* = 3.27 mT, it results in a stimulation frequency of 10 KHz (100 times higher than the highest frequency tested in this work) to get a temperature enhancement of 0.06°C s^−1^.

The theoretical model was then experimentally validated by measuring the increase of temperature using an infrared thermo-camera during the electromagnetic stimulations with NPs. No temperature variations were observed, thus revealing a good agreement with the model.

Finally, the results collected allowed us to exclude dissolution of NPs and hyperthermia effects from the mechanisms responsible for the magnetically triggered IBU release. This conversely can be explained by a faster mechanical movement/vibration (i.e. shaking and flipping) of FeHAs at higher frequency of the EMF, prompting the detachment of IBU molecules from their surface.

### 3.5. Analysis of *in vitro* cardiac activity with a magnetic/electro-magnetic bioreactor device

**Figure 5.**
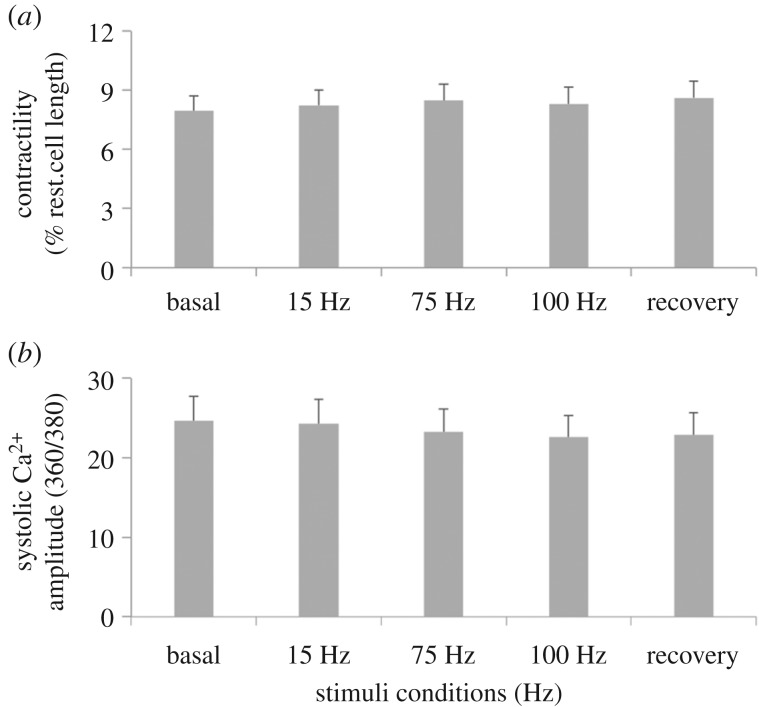
Cardiomyocyte activity. Contractility (*a*) and Ca^2+^ transients (*b*) in adult cardiomyocytes at different electromagnetic stimulations. All values are reported as mean ± s.e.m.

Our next question was to assess whether the identified MEBD configuration necessary to achieve efficient drug release from FeHAs was compatible with the physiologic activity of cardiomyocytes (CMCs). To this end, sarcomere shortening, an index of contractile properties as well as peak height of Ca^2+^ transients, an index of intracellular Ca^2+^ concentration, were evaluated *in vitro* from isolated adult CMCs exposed to short-term EMF stimulation. Notably, both parameters were found unaltered at any of the ELM stimulations tested supporting the biocompatibility of the MEBD with the biological system (figure 5). Altogether, these data provide evidence that the developed MEBD is able to generate a range of electromagnetic stimulations compatible with physiological cardiac function.

### 3.6. Analysis of *in vivo* cardiac activity with a magnetic/electro-magnetic bioreactor device

**Figure 6.**
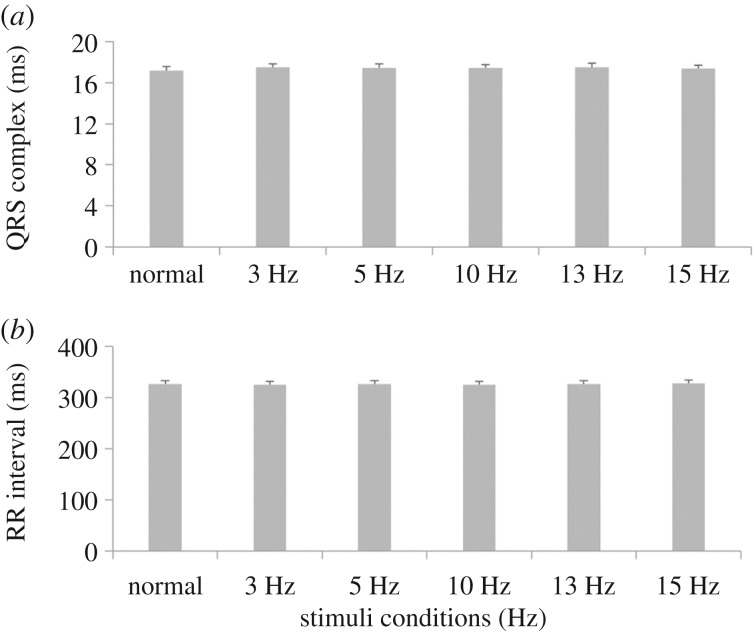
*In vivo* cardiac ECG. QRS complex (*a*) and RR interval (*b*) durations at different electromagnetic stimulations. All values are reported as mean ± s.e.m.

The effects of the low EMF stimulations on cardiac activity were also evaluated *in vivo* on ECG recorded from rats. In particular, QRS complex and RR interval data from continuous multiple frequency stimulation were statistically analysed (figure 6). No significant effects were found on the RR interval, thus showing that the heart auto-rhythmicity was not affected by the pulsed EMF. Moreover, measurements of the QRS complex showed no significant effects, demonstrating that ventricular activation is unchanged after MEBD magnetic stimulation. In line with this, all animals were healthy after each experimental procedure without showing any observational issues or discomfort. We introduced 30 s recovery between stimuli where the heart was left to beat without EMF stimulation. This seems to be beneficial for cardiac performance as indexed by the very small R-R variation.

## 4. Discussion

The objective of this study was to experimentally validate the feasibility of controlled drug release from superparamagnetic FeHAs under a MEBD-mediated low-pulsed EMF stimulus integrated within a fluidic circuit. The effects of this EMF stimulation as well as FeHA administration were also monitored both *in vitro* and *in vivo* in terms of biocompatibility with the cardiac setting. The results obtained pave the way for the possible use of FeHAs and MEBD for the treatment of CVDs, where external magnetic stimuli might be applied to selectively trigger the release of drugs from the nano-carriers at the heart level and without altering the physiological function of the myocardium. FeHAs were selected among the existing MNPs because they have a lower content of iron compared to SPIONs (about 10 wt% versus 71 wt%) [36] and for their potential cardio-compatibility, as recently shown by our group for the undoped non-magnetic CaPs [4,6]. In fact, without promoting toxicity or interfering with any functional properties of cardiomyocytes, CaPs were shown *in vitro* and *in vivo* to safely and successfully cross the cardiomyocyte cellular membrane and release bioactive molecules (i.e. microRNAs and peptides) inside the cell [4,6]. In line with this study, we found here that FeHAs, similarly to undoped non-magnetic CaPs, possess good biocompatibility with cardiac cells and internalize through a clathrin/dynamin-mediated process.

The use of low invasive external EMFs for the manipulation of MNPs as well as for field-controlled drug delivery and release applications is nowadays an important topic [7,15,20,40]. One of the most common strategies to activate drug release by alternating EMF, mainly in cancer therapy, consists of the embedding of MNPs in thermo-responsive polymers [8,41]. In these cases, the effect of EMF that is typically in the kilohertz range not only favours drug release but also induces tumour cell death through a local MNP-mediated temperature increase. As the aim of this work was to set the basis for a new treatment for CVDs, here the MEBD was conceived to generate a much lower field intensity to avoid any possible damage to cells and tissues caused by intense EMF stimulus and hyperthermia. The magnetically induced drug release from FeHAs has been evaluated *in vitro* in a fluidic system mimicking the circulatory micro-environment. IBU has been adopted as a tool drug to monitor the effect of both EMF frequencies and flux on the time-dependent drug release. Interestingly, the kinetics of IBU release is significantly affected by the frequencies applied and in the absence of any significant hyperthermia effect, finally suggesting the possibility to properly control drug release according to the magnetic stimulation provided.

Although the physico-chemical interaction of the drug with FeHAs could vary considerably by changing the loaded molecule, the drug release enhancement by increasing the stimulating frequencies up to 100 Hz can be reasonably extended to other drugs/bioactive molecules.

The use of EMF and MNPs to provide non-invasively and spatially localized drug release is still in its infancy and these technologies are not rapidly progressing to the clinics due to high experimental complexity, different field exposure protocols and related functional parameter variations. In fact, the approaches based on the EMF still have to be carefully validated to assess that no side effects can be derived on cell activity and tissue functionality. In fact, although, on the one hand, *in vitro* studies showed that the electrical stimulation does not hamper physiological cellular activity (e.g. cellular growth, proliferation [42,43] and the differentiation of murine and rat neuronal cells [13,28], on the other hand several concerns have been raised around possible alterations of the electrophysiological properties of cardiac cells exposed to EMF. Notably, a number of studies have reported that an external EMF application may change Ca^2+^ metabolism, which is of vital importance in the cardiac system [29,44,45]. This is due to the voltage-dependent behaviour of Ca^2+^ channels and thus Ca^2+^, which is responsible for cardiac contractions and rhythmicity [46]. For example, it was shown that an extremely low EMF can enhance the intracellular Ca^2+^ transients in CMCs [47]. Other studies reported no damage ascribable to the application of a low-frequency pulsing EMFs on rats with acute experimental myocardial infarcts [48]. In particular, continuous exposure to 3 mT, 75 Hz-pulsed extremely low-frequency EMFs decreased the amount of permanently injured myocardium after ligation of the left anterior descending coronary artery in rats [48].

Recently, some MEBDs have been proposed to electrically/magnetically stimulate cells in a systematic and reproducible manner; however, their operating conditions are in some case limited in frequency ranges and current intensities, thus reducing their flexibility [49,50].

Here, the activity of both primary CMCs *in vitro* and heart *in vivo* has been observed by adopting different frequencies of magnetic stimulation, showing that EMFs with intensities in the range of 3–4 mT did not affect CMC physiology regarding contractile properties and Ca^2+^ transients (*in vitro*) and QRS complex and RR variability (*in vivo*), these being physiological parameters constant through the entire experiment for the overall frequency range adopted.

## 5. Conclusion

In conclusion, it was proven that FeHAs could be used as functional cardio-compatible nano-carriers for the magnetically assisted delivery of bioactive molecules under fluid-flow conditions resembling those of the cardiovascular environment. A custom-made MEBD has been developed as a promising apparatus for drug delivery applications, where MNPs might be used for a controlled drug release in the cardiac district with no heat generation. Frequencies lower than 100 Hz were shown to not alter CMC properties such as contractile properties and Ca^2+^ transients, and no ECG alterations were found *in vivo*.

These results open a new scenario in the field of a magnetically assisted drug delivery system for the treatment of CVDs. Furthermore, owing to its versatility, we foresee that the MEBD proposed here might be applied in different clinical applications depending on the MNPs-bioactive molecule system used.

## Supplementary Material

Figures S1 - S4 and Table S1

## Data accessibility

The datasets supporting this article have been uploaded as part of the electronic supplementary material.
