# Supplementary material for: A combined low-frequency electromagnetic and fluidic stimulation for a controlled drug release from superparamagnetic calcium phosphate nanoparticles: potential application for cardiovascular diseases
Source: J R Soc Interface. 2018 Jul 11;15(144):20180236. doi: 10.1098/rsif.2018.0236 (PMC6073647; doi:10.1098/rsif.2018.0236)
Supplement: Figures S1 - S4 and Table S1 [file rsif20180236supp1.docx]

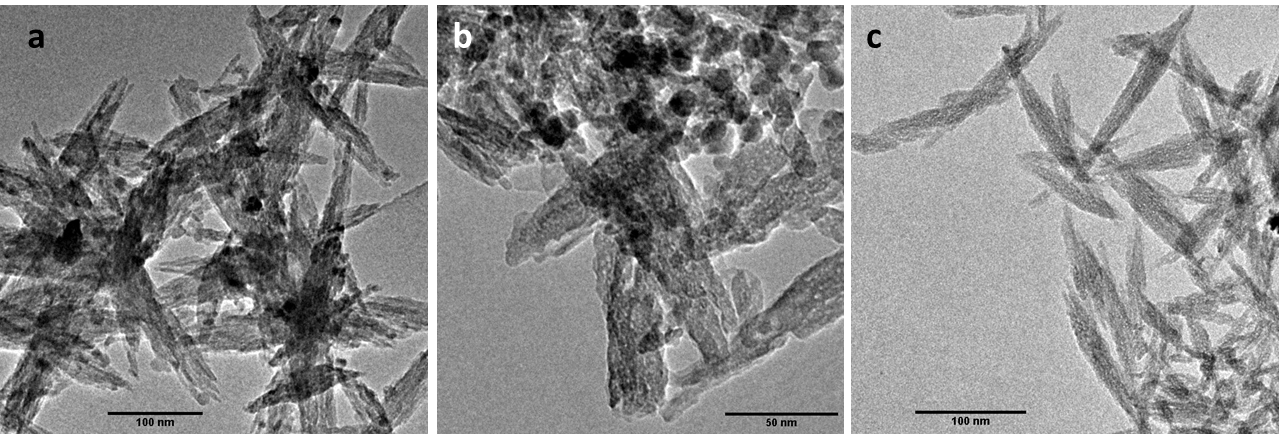


**Figure S1**. Transmission Electron Microscopy (TEM) images of FeHAs (a, b) at two different magnifications and HAs (c).

**Figure S2.** Hydrodynamic diameter distributions of FeHA and HA in HEPES buffer 0.1 M, pH 7.4.

Electro-stimulating bioreactor development

The magnetic flux density *B(x)* along the axis crossing the center of coils is derived by the Biot-Savart law (eq.1); it is proportional to the current *I* flowing through the coils and the number *N* of turns in each coil placed at a certain distance *h*:

$\vec{B}=\frac{N\mu_{0}Ia^{2}}{2}\frac{\vec{\mu}_{z}}{\left[ a^{2}+d^{2} \right]^{\frac{3}{2}}}+\frac{N\mu_{0}Ia^{2}}{2}\frac{\vec{\mu}_{z}}{\left[ a^{2}+\left( h-d \right)^{2} \right]^{\frac{3}{2}}}$ (1)

Where *a* is the radius of the coils, *µ_0_* is the permeability of free space (4π 10^-7^Tm/A), *d* = 0 and *d* = *h* correspond to the center of coils.

The EMF was generated by waveforms generator (LecroyWavestation2022) connected to a current amplifier (QD model4480). The generator has two output channels and allows generating waves of type sine, square, ramp, and pulse. The current amplifier has 4 output channels, impedance that can vary from 4 to 8 ohms depending on the type of configuration, and a maximum output power of 80 W per channel. The amplifier is connected with a copper wire to a resistor block. The current flowing through the coils is proportional to the applied voltage, while the intensity of the EMF is directly proportional to the current. The resistance of the coils has been measured as follow:

$R=\frac{\rho L}{A}$ (2)

Where ρ is the conductivity of copper wire (1,69 10^-8^ Ohm x meter), while *L* and *A* are the length and section of the wire, respectively.

The resistor block has been then realized by connecting 4 resistors in series, each of 1Ω and 50 W to avoid the overheating of the circuit. The resistor block has been then connected to the Helmholtz coils.

Technical data of the electro-stimulating bioreactor are summarized in Table S1.

**Table S1.** Technical data of each component of the MEBD.

| **Generator** | |
| --- | --- |
| Voltage Input | ±6V |
| Amplitude (CH1) | 4mVpp-6Vpp |
| Frequency range | 1µHz to 25MHz |
| Power supply | 100 – 240 V/AC 50/60 Hz, 100 – 120 V/AC 400 Hz |
| Output Impedance | 50 Ω |
| **Coils** | |
| Radius | 50 mm |
| Distance between them | 50 mm |
| Number of turns | 50 |
| Diameter of copper threads | 1,18 mm |
| Resistance | 0,48 Ohm |
| Inductance | 1 mH |
| **Amplifier** | |
| Power consumption | 80 W |
| Frequency range | 20Hz to 50KHz |
| Power supply | 100-120VAC or 220-240VAC |
| Output Impedance | 4-8 Ω |
| Distortion | <0.1% THD, 20Hz - 20kHz |
| Input Sensitivity | 0.775 Vmrs |
| Working range (V) | 1-40V |

Adsorption of IBU on FeHAs

The adsorption study was organized in two steps aiming, firstly, at inspecting the kinetics of adsorption, and secondly, at determining the adsorption isotherms.

In the first stage, the adsorption kinetics of IBU on FeHAs and HAs were investigated. The kinetic overview was made possible by following the amount of IBU adsorbed (Q_ads_) as a function of contact time between the NPs and the drug. The evolution of Q_ads_ (given in μmol IBU m^-2^ NPs) versus time is plotted on Fig. S1 in the case of a constant IBU concentration of about 50 mmol L^-1^. The specific surface area of FeHA and HA, measured using a BELSORP mini II apparatus (Microtrac, Krefeld, Germany) (BET method based on nitrogen adsorption) were 90 ± 9 and 85 ± 9 m^2^ g^-1^, respectively.





**Figure S3**. Adsorption kinetics of IBU on (▪) FeHAs and (▫) HAs.

These data indicated that in both cases the adsorbed amount increased as a function of time with a first rapid rise followed by a progressive stabilization: up to around 6 μmol IBU m^-2^ NPs (about 1.2 mg m^-2^) in the case of HA compared to an higher value of ca. 9 μmol IBU m^-2^ NPs (about 1.9 mg m^-2^) for FeHA. The shape of these kinetic curves points to a rather rapid adsorption of the IBU molecules slightly faster for FeHA (around 60 min) than for HA (around 150 min).

In the second stage, using a selected contact time of 150 min the adsorption isotherms of IBU on both FeHA and HA have been analyzed by performing experiments with increasing IBU concentrations (1 to 75 mg ml^-1^). Fig. S2 reports the obtained isotherms for Q_ads_ expressed in μmol IBU m^-2^ NPs and C_eq_ (equilibrium concentration) in mmol L^-1^.





**Figure S4**. Adsorption isotherms of IBU on (▪) FeHAs and (▫) HAs. Separate points are the experimental data; dotted lines indicate Sips fits of isotherm data.

The two isotherms exhibited a similar shape revealing a comparable mechanism of adsorption. The isotherm curves showed a first steep increase of the amount of IBU adsorbed at an equilibrium concentration of up to C_eq_ about 100 mmol L^-1^, followed by stabilization at higher concentrations. This general tendency points to an adsorption behavior with a progressive filling of surface sites and towards a maximal degree of coverage. This type of evolution is noticed in particular for the Langmuir model and for the more general equation of Sips (Langmuir–Freundlich) [[1](#_ENREF_1)]:

$$Q_{ads}=Q_{m}\cdot\frac{K_{s}\cdot C_{eq}^{m}}{1+K_{s}\cdot C_{eq}^{m}}$$

where Q_m_ designates the maximal adsorption coverage, C_eq_ is the equilibrium concentration of IBU for the considered data-points, ‘‘m’’ is the Sips exponent, and K_S_ is the Sips constant [[1](#_ENREF_1)].

A mathematical analysis indicates that the adsorption data for both NPs can be well fitted with the Sips model (R^2^ = 0.9786 for HA and R^2^ = 0.9729 for FeHA). The Sips parameters that were found are the following: Q_m_ = 23.51 ± 1.76 μmol IBU m^-2^ FeHA, m = 2.65 ± 0.60, K_s_ = 1.88·10^-5^ ± 4.35·10^-5^ (for C_eq_ expressed in mmol L^-1^) in the case of FeHA; Q_m_ = 31.51 ± 1.38 μmol IBU m^-2^ HA, m = 4.88 ± 1.38, K_s_ = 3.53·10^-9^ ± 1.93·10^-8^ (for C_eq_ expressed in mmol L^-1^) in the case of HA. Despite a non-negligible uncertainty we can assume that FeHA has an stronger affinity to IBU respect to HA (according to the values of K_s_) but a lower maximum degree of loading. The value of ‘‘m’’ appeared to be in both cases noticeably distinct from unity, thus suggesting a neat departure from the standard Langmuirian case. A value of ‘‘m’’ greater than 1 can suggest the existence of significant positive interactions (molecular cooperativity) of adsorbed molecules among each other [[2](#_ENREF_2)].

A recent work about the interaction of IBU with nanocrystalline HA pointed out that this occurs by a hydrogen bond between the hydroxyl group of IBU and one of the hydroxyl groups of the HA, along with an interaction between the carbonyl group of IBU and a Ca ion of HA [[3](#_ENREF_3)].

**References**

1 Iafisco, M., Drouet, C., Adamiano, A., Pascaud, P., Montesi, M., Panseri, S., Sarda, S., Tampieri, A. 2016 Superparamagnetic iron-doped nanocrystalline apatite as a delivery system for doxorubicin. *Journal of Materials Chemistry B*. **4**, 57-70. (10.1039/c5tb01524c)

2 Koopal, L. K., van Riemsdijk, W. H., de Wit, J. C. M., Benedetti, M. F. 1994 Analytical Isotherm Equations for Multicomponent Adsorption to Heterogeneous Surfaces. *J. Colloid Interface Sci.* **166**, 51-60. (https://doi.org/10.1006/jcis.1994.1270)

3 Ryabenkova, Y., Jadav, N., Conte, M., Hippler, M. F. A., Reeves-McLaren, N., Coates, P. D., Twigg, P., Paradkar, A. 2017 Mechanism of Hydrogen-Bonded Complex Formation between Ibuprofen and Nanocrystalline Hydroxyapatite. *Langmuir*. **33**, 2965-2976. (10.1021/acs.langmuir.6b04510)
